# Hepatoprotective Effect of *Morchella* Mycelia Polysaccharides on Alcoholic Liver Injury and Its Mechanism Involving the Modulation of NOD‐Like Receptor Signaling Pathway

**DOI:** 10.1002/fsn3.71483

**Published:** 2026-01-23

**Authors:** Xinyu Hu, Qinghu Duan, Leran Ma, Tianyuan Zhang, Mengdan Zhai, Zewei Chen, Wangqi Li, Kaiwen Huang, Yan Ma, Yuchen Zhang, Zhen Wang

**Affiliations:** ^1^ College of Biology and Food Engineering Huanghuai University Zhumadian China; ^2^ Xinyang City Academy of Agricultural Sciences Xinyang Henan China; ^3^ School of Life Sciences and Technology Henan Medical University Xinxiang China; ^4^ Agricultural Genomics Institute at Shenzhen, Chinese Academy of Agricultural Sciences Shenzhen China; ^5^ Institute of Edible Fungi, Shanghai Academy of Agricultural Sciences; Key Laboratory of Edible Fungi Resources and Utilization (South), Ministry of Agriculture and Rural Affairs, P. R. China; National Engineering Research Center of Edible Fungi; National R&D Center for Edible Fungi Processing; Key Laboratory of Agricultural Genetics and Breeding of Shanghai Shanghai China

**Keywords:** alcoholic liver injury, *Morchella*, Nlrp3, polysaccharides, transcriptome

## Abstract

Alcoholic liver disease (ALD) is one of the leading causes of preventable liver‐related morbidity and mortality globally. Bioactive polysaccharides exhibit substantial potential as functional foods and therapeutic agents for the prevention and treatment of alcoholic liver injury (ALI). *Morchella*, an edible and medicinal fungus, contains polysaccharides with diverse biological activities. This study aimed to evaluate the protective effects of *Morchella* mycelium polysaccharides (MP) against alcohol‐induced liver injury and elucidate the underlying mechanisms. The MP was isolated from the *Morchella* mycelium using water extraction–ethanol precipitation. Its primary component was glucose (96.555%), with a weight‐average molecular weight of 5.7 kDa and an α‐glycosidic configuration. These characteristics indicated a highly homogeneous polysaccharide structure. Research findings demonstrated that the MP significantly reduced serum alanine aminotransferase (ALT) and aspartate aminotransferase (AST) levels, improved lipid metabolism (evidenced by decreased triglyceride and cholesterol levels), and restored the histopathological structure of the mouse liver. Mechanistically, the MP alleviated oxidative stress by enhancing the activity of antioxidant enzymes (superoxide dismutase, catalase, and glutathione) and inhibiting lipid peroxidation (indicated by reduced malondialdehyde levels). Transcriptomic analysis further revealed the anti‐inflammatory mechanism of MP. It downregulated the expression of *Ifi16*, *Pycard*, and *Nlrp3* by suppressing the Nlrp3 inflammasome in the NOD‐like receptor signaling pathway. This suppression subsequently inhibited *pro‐Casp1* activation and the pyroptosis of hepatocytes. Additionally, the MP upregulated the antimicrobial peptide *Camp*, highlighting its dual functions in anti‐inflammation and intestinal barrier protection. Collectively, these results suggest that *Morchella* mycelia polysaccharide, as a potent natural compound, holds significant promise for combating alcohol‐induced liver injury.

## Graphical abstract

The *Morchella* mycelium polysaccharide (MP), a homogeneous α‐glucan (96.555% glucose, 5.7 kDa), protects against alcohol‐induced liver injury in mice. It reduces serum ALT/AST, improves lipid metabolism, and enhances antioxidants and inhibiting lipid peroxidation. Mechanistically, the MP suppresses *Nlrp3* inflammasome‐mediated inflammation/pyroptosis and upregulates *Camp* for intestinal barrier protection.

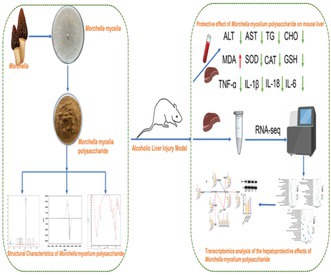

**Abbreviations**
ACLF: acute chronic liver failure
AH: alcohol‐related hepatitis
ALD: alcoholic liver disease
ALI: alcoholic liver injury
ALT: alanine aminotransferase
AST: aminotransferase
CAT: catalase (CAT)
CHO: cholesterol
DEGs: differentially expressed genes
ELISA: enzyme‐linked immunosorbent assay
GO: gene ontology
GSH: reduced glutathione (GSH)
HCC: hepatocellular carcinoma
HMW: high‐molecular‐weight
HPGPC: high‐performance gel permeation chromatography
HPLC: high‐performance liquid chromatography
IL‐18: interleukin‐18
IL‐1β: interleukin‐1β
IL‐6: interleukin‐6
KEGG: Kyoto Encyclopedia of Genes and Genomes
LMW: low‐molecular‐weight
MDA: malondialdehyde (MDA)
Mw: molecular weight
PDI: polydispersity index
qPCR: fluorescent quantitative PCR
ROS: reactive oxygen species
SOD: superoxide dismutase
TC: triglycerides
TNF‐α: tumor necrosis factor‐α

## 1 Introduction

Alcoholic liver disease (ALD) is the primary cause of preventable morbidity and mortality related to the liver on a global scale (Li et al. 2020). The ALD spectrum covers steatosis, steatohepatitis, fibrosis, cirrhosis, and hepatocellular carcinoma (HCC). It also includes alcohol‐related hepatitis (AH)—a distinct manifestation that can progress to severe acute chronic liver failure (ACLF) (Korkida et al. 2024). Globally, harmful alcohol use causes 3 million deaths annually. This accounts for 5.3% of all fatalities and 13.5% of deaths among people aged 20–39 (Sempokuya et al. 2022). Harmful alcohol consumption is a significant risk factor for liver injury and alcoholic liver disease (Li et al. 2020). Moreover, growing evidence indicates that the health benefits associated with alcohol consumption are minimal to nonexistent, and any level of drinking has the potential to cause harm (Nóbrega et al. 2024).

In the human body, the liver serves as the primary organ responsible for alcohol catabolism and detoxification. Chronic or excessive alcohol consumption can lead to alcoholic liver injury (ALI) (Su et al. 2023). Ingested alcohol is absorbed through the gastrointestinal tract, with less than 10% being excreted via breath, sweat, and urine. Consequently, over 90% of the absorbed alcohol enters the bloodstream and is ultimately transported to the liver via the portal vein (Xiong et al. 2024). In the liver, alcohol is metabolized via both oxidative and non‐oxidative pathways, with the oxidative pathway being the predominant route (Han et al. 2023). Within hepatocytes, alcohol dehydrogenase converts alcohol into acetaldehyde—a highly toxic compound with a short half‐life. Acetaldehyde is then further metabolized into acetate by acetaldehyde dehydrogenase (Sun et al. 2022). Chronic exposure to high doses of alcohol can induce the upregulation of CYP2E1, resulting in accelerated metabolism (Zhao et al. 2023). This process generates elevated levels of reactive oxygen species (ROS) within hepatocytes, reducing intracellular antioxidants such as S‐adenosylmethionine and glutathione (Song et al. 2024). The non‐oxidative pathways contribute minimally to overall alcohol metabolism. Minor quantities of alcohol are non‐oxidatively transformed into various endogenous metabolites by distinct enzymes (Han et al. 2023). Timely and effective management of alcohol‐related disorders is a critical foundation for enhancing the quality of life among individuals with alcoholism. To date, alcohol dehydrogenase inhibitors and opioid receptor antagonists have been predominantly employed in the treatment of ALD. Nevertheless, prolonged administration of these medications may induce a range of adverse reactions in patients, including drug dependency, nausea, dermatological issues, dizziness, and leukopenia (Cheng et al. 2022). Thus, exploring the therapeutic potential of natural, edible fungi with potent antioxidant and anti‐inflammatory properties represents a safer and more health‐promoting approach to addressing ALD.

*Morchella esculenta* (L.) Pers. ( *M. esculenta* ) is a rare species of edible and medicinal fungi (Hu et al. 2024). The primary bioactive component of *M. esculenta* is its polysaccharides. Research indicates that these polysaccharides exhibit a range of biological activities, including enhancing immune function, exhibiting anti‐tumor properties, and demonstrating antioxidant effects (Li et al. 2023). Studies have demonstrated that various bioactive polysaccharides provide significant protective effects against ALI. These effects are primarily achieved through the activation of enzymes involved in alcohol metabolism, the attenuation of damage caused by oxidative stress, the regulation of cytokine levels, the inhibition of hepatocyte apoptosis, and the modulation of intestinal microbiota (Su et al. 2023). Bioactive polysaccharides have significant potential as functional foods and medicinal agents for the prevention and treatment of ALI. Similar to other natural polysaccharides, *Morchella* polysaccharides are classified as dual‐use compounds with both medicinal and nutritional properties. Nevertheless, the research and development of *Morchella* polysaccharides remain relatively underexplored, particularly concerning their protective effects against ALI. Therefore, this study established the mouse model of ALI and an experimental group treated with MP. Through the detection of biochemical indicators in serum and liver homogenate, observation of liver tissue pathology, and analysis of the protective mechanisms of *Morchella* polysaccharides against ALI using transcriptomics, this research aims to identify a novel liver protection pathway. This work provides a crucial theoretical foundation for the development of more efficacious liver disease treatments and therapeutic strategies, while also promoting advancements in morel cultivation, processing, and associated industries.

## 2 Materials and Methods

### 2.1 Extraction of Polysaccharide From Morchella Mycelium

The mycelium of *Morchella*, preserved in the laboratory, was selected as the experimental sample. The liquid culture of the *Morchella* mycelium was subjected to repeated washing with distilled water, followed by drying at 60°C until a constant weight was achieved. The quantitatively dried *Morchella* mycelium was subsequently weighed, and an appropriate volume of ultra‐pure water was added based on a material‐to‐solvent ratio of 1:40 (g/mL). The water bath temperature was maintained at 85°C for a duration of 90 min. Following the water bath treatment, the mixture was subjected to centrifugation at 6000 rpm for 15 min. Following centrifugation, the supernatant was collected and concentrated. Subsequently, anhydrous ethanol was added in a ratio of four volumes to one volume of the concentrated supernatant, mixed thoroughly, and the mixture was incubated at 4°C for 24 h. Upon completion of the alcohol precipitation period, the sample was centrifuged at 6000 rpm for 20 min. The supernatant was discarded, and the precipitate was collected, yielding the crude polysaccharide. The crude polysaccharide was dissolved to form a 15 mg/mL solution and subsequently purified using a DEAE Sephadex A‐25 ion‐exchange column (Sigma‐Aldrich, Shanghai, China). Elution was performed at a rate of 2 mL/min with NaCl (Sinopharm Chemical Reagent Co. Ltd., Shanghai, China) solutions of 0.3, 0.9, 1.5, and 2.0 mol/L. The eluted fractions were collected and lyophilized to yield the final polysaccharide samples (Tian et al. 2024).

### 2.2 Identification of Monosaccharide Components of Polysaccharides

The purified polysaccharide was subjected to complete hydrolysis to convert it into monosaccharides. The hydrolyzed sample solution was adjusted to a fixed volume, filtered through a 0.45 μm filter membrane, and subsequently utilized as the test product solution. A precise amount of monosaccharide standard (Sinopharm Chemical Reagent Co. Ltd., Shanghai, China) was weighed and dissolved in ultra‐pure water to prepare a monosaccharide standard stock solution with a concentration of 1.0 mg/mL. Appropriate volumes of each monosaccharide standard stock solution were then accurately pipetted and diluted with the mobile phase to generate a series of mixed standard solutions at concentrations of 0.1, 0.2, 0.5, 0.8, and 1.0 mg/mL. High‐performance liquid chromatography (HPLC) analysis was performed by injecting 20 μL aliquots of these mixed standard solutions at varying concentrations, and the resulting chromatograms were recorded. Each concentration was injected in triplicate, and the average peak area was calculated. Subsequently, 20 μL of the test product solution was injected into the HPLC system (Shimadzu, Shanghai, China), and the chromatogram was recorded with three parallel injections. Based on the retention times of the monosaccharide standards, the chromatographic peaks in the test product's chromatogram were qualitatively identified to determine the types of monosaccharides present in the sample. A standard curve was constructed using the concentrations of the mixed standard solutions as the x‐axis and their corresponding peak areas as the y‐axis. The concentrations of individual monosaccharides in the sample were determined from their respective peak areas using the standard curve, and the content of each monosaccharide in the polysaccharide sample was subsequently calculated based on the dilution factor and the sample weight (Wang et al. 2017).

### 2.3 Determination of the Relative Molecular Mass of Polysaccharides

A 50 mg sample of polysaccharides was accurately weighed and transferred into a 10 mL volumetric flask. The sample was dissolved in the mobile phase (0.1 M sodium nitrate solution) (Sinopharm Chemical Reagent Co. Ltd., Shanghai, China), and the volume was adjusted to the calibration mark. The solution was thoroughly mixed and subsequently filtered through a 0.45 μm membrane filter. The resulting filtrate was collected for analysis. Appropriate quantities of Dextran T‐300, T‐150, T‐10, T‐5, and glucose (Sinopharm Chemical Reagent Co. Ltd., Shanghai, China) were accurately weighed, individually dissolved in the mobile phase, and diluted to the required volumes to prepare standard solutions of defined concentrations. These standard solutions were also filtered through 0.45 μm membrane filters prior to analysis. The Waters 2695 high‐performance liquid chromatograph (Waters, Milford, Massachusetts, USA) was employed for analysis. The column temperature was maintained at 40°C, and the mobile phase, consisting of 0.1 M sodium nitrate solution (Sinopharm Chemical Reagent Co. Ltd., Shanghai, China), was delivered at a flow rate of 0.5 mL/min. A series of standard solutions were sequentially injected, and the retention time of each standard was recorded. A calibration curve was constructed by plotting the logarithm of the molecular weight (lgM_W_) against the corresponding retention time (tR). Subsequently, the sample solution was injected, and its retention time was determined. The relative molecular mass of the polysaccharide was then calculated by interpolation using the established calibration curve (Meng et al. 2023).

### 2.4 Infrared Spectroscopy Analysis of Polysaccharides

The functional groups present in the polysaccharide samples were analyzed using the KBr pellet method. Briefly, 2 mg of the fully dried polysaccharide sample was accurately weighed and uniformly mixed with 200 mg of spectroscopic‐grade KBr. The mixture was then compressed into a transparent pellet, with a pure KBr pellet employed as the background reference. Fourier‐transform infrared (FT‐IR) spectra were recorded on a Nicolet iS‐10 spectrometer (Thermo Scientific, Waltham, USA) over the wavenumber range of 400–4000 cm^−1^, with a resolution of 4 cm^−1^ and an accumulation of 32 scans (Wang et al. 2020).

### 2.5 Development of the Murine Model for Alcoholic Liver Injury

Twenty‐four healthy male mice (SPF‐grade C57BL/6 mice, 8 weeks old, weighing 20–22 g) were randomly divided into four groups (*n* = 6): the blank control group (Control), the model group (Model), the positive drug administration group (Sil), and the *Morchella* mycelium polysaccharide group (MP). The blank control group received intragastric administration of normal saline (Sinopharm Chemical Reagent Co. Ltd., Shanghai, China) at a dose of 0.1 mL/10 g body weight twice daily (at 9:00 AM and 4:00 PM) for seven consecutive days. The other groups were administered 53‐proof liquor (Huanghuai Wheat Aroma‐Type Baijiu, College of Biology and Food Engineering, Huanghuai University, Zhumadian, China) at a dose of 12 mL/kg once daily (at 9:00 AM). Seven hours post‐administration, the model group received an intragastric dose of 0.1 mL/10 g normal saline, the positive drug administration group was given silymarin (Sinopharm Chemical Reagent Co. Ltd., Shanghai, China) at 70 mg/kg, and the treatment group received *Morchella* polysaccharide at 200 mg/kg (selected from previous studies on fungal polysaccharides), all for seven consecutive days (Teng et al. 2023). The body mass of each mouse was recorded daily at a consistent time. On the final day of the experimental period, the mice underwent an overnight fast, after which blood samples were collected via cardiac puncture under anesthesia. Subsequently, the mice were humanely euthanized, and their livers were promptly excised and weighed. A single lobe of the liver from each mouse was fixed in 4% paraformaldehyde solution (Servicebio, Wuhan, China), while the remaining tissue was flash‐frozen in liquid nitrogen and stored at −80°C for further analysis. The experimental design and procedural methodologies of this study were reviewed and endorsed by the Experimental Animal Ethics Committee of Huanghuai University (Approval No. HHU20220008).

### 2.6 Detection of Biochemical Indices

The collected blood samples were maintained at room temperature for 2 h, followed by centrifugation at 3000 rpm at 4°C for 15 min. The serum was subsequently isolated, and the levels of aspartate aminotransferase (AST), alanine aminotransferase (ALT), triglycerides (TC), and cholesterol (CHO) were quantified using an automated biochemical analyzer (Servicebio, Wuhan, China). The liver tissue was weighed and homogenized in a 1:9 weight‐to‐volume ratio (mg: μL) with physiological saline (0.9%) (Sinopharm Chemical Reagent Co. Ltd., Shanghai, China). The liver tissue was mechanically homogenized in an ice bath, and the homogenate was centrifuged at 3000 rpm for 10 min to obtain the supernatant. The levels of malondialdehyde (MDA), reduced glutathione (GSH), superoxide dismutase (SOD), and catalase (CAT) were measured using specific detection kits. Additionally, the concentrations of interleukin‐6 (IL‐6), interleukin‐1β (IL‐1β), interleukin‐18 (IL‐18), and tumor necrosis factor‐α (TNF‐α) in the liver homogenate were determined using the enzyme‐linked immunosorbent assay (ELISA) (Servicebio, Wuhan, China).

### 2.7 Observation of Pathological Sections

Liver specimens were fixed in 4% paraformaldehyde (Servicebio, Wuhan, China), dehydrated using gradient ethanol (Sinopharm Chemical Reagent Co. Ltd., Shanghai, China), and subsequently processed for paraffin embedding and sectioning. Subsequently, pathological changes in liver tissues were evaluated via hematoxylin and eosin (HE) staining and examined under an optical microscope (Teng et al. 2023).

### 2.8 Transcriptome Sequencing Analysis

Total RNA from liver tissue samples was extracted using the Trizol method (Hu et al. 2021). The concentration and purity of the RNA were assessed using a Thermo Scientific Nanodrop 2000 spectrophotometer (Thermo Scientific, Waltham, USA). RNA samples with a concentration of ≥ 1 μg were selected for library preparation using the NEBNext Ultra II RNA Library Prep Kit for Illumina (New England Biolabs Inc., Ipswich, USA). The quality of the library was evaluated using an Agilent 2100 Bioanalyzer (Agilent, Beijing, China). The total concentration of the library was determined with Picogreen dsDNA Quantitation Reagent (Thermo Scientific, Waltham, USA), while the effective concentration of the library was quantified via Fluorescent quantitative PCR (qPCR). Following a series of dilutions and quantifications of the pooled libraries, paired‐end sequencing (PE150) was conducted on Illumina sequencers (Illumina, San Diego, USA) (Hu et al. 2024).

The samples were sequenced to acquire the raw data, after which Fastp v0.22.0 was employed to filter the raw data, resulting in clean data (Chen et al. 2023). An index of the reference genome (http://www.ensembl.org/Mus_musculus/Info/Annotation) was constructed using HISAT2 v2.1.0 (Kim et al. 2019). HTSeq v0.9.1 was utilized to quantify the read counts of each gene as the raw measure of gene expression (Liu et al. 2022). FPKM (Fragments Per Kilobase of transcript per Million mapped reads) normalization was applied to standardize the expression levels. DESeq2 v1.38.3 was then utilized to identify differentially expressed genes (DEGs) between the comparison groups (Liu et al. 2021). The criteria for screening DEGs were as follows: expression difference multiple |log_2_ Fold Change| > 1, with a significant *p* < 0.05. Furthermore, Gene Ontology (GO) and Kyoto Encyclopedia of Genes and Genomes (KEGG) enrichment analyses were performed using cluster Profiler v4.6.0.

### 2.9 Detection of Fluorescence Quantitative PCR (qPCR)

Reverse transcription was performed using SynScriptIII RT SuperMix (Tsingke, Wuhan, China) for subsequent qPCR. The resultant cDNA was diluted threefold and utilized as the template for qPCR amplification with ArtiCanCEO SYBR qPCR Mix (Tsingke, Wuhan, China). The composition of the amplification system is as follows: ArtiCanCEO SYBR qPCR Mix (10 μL), forward primer (10 μM, 0.8 μL), reverse primer (10 μM, 0.8 μL), cDNA template (1 μL), and ddH2O (7.4 μL). Amplification was performed according to the following cycling conditions: initial denaturation at 95°C for 5 min, followed by 40 cycles of denaturation at 95°C for 15 s, annealing at 60°C for 20 s, and extension at 70°C for 20 s. In this qPCR analysis, *Gapdh* was employed as the internal reference gene (Wang et al. 2023). The specific primer sequences are listed in Table S1.

### 2.10 Weighted Gene Co‐Expression Network Analysis (WGCNA)

The DEG co‐expression network was constructed using the WGCNA package (version 1.69) in R software (Hu et al. 2024). The adjacency matrix was generated by calculating the Pearson correlation coefficients for all DEGs. A network with an approximately scale‐free topology was established based on the criterion of selecting a soft‐threshold power *β* set to 12 (*R* ^2^ = 0.8).

### 2.11 Western Blot Analysis

The mouse liver tissue was washed with PBS, and total protein was subsequently extracted using RIPA lysis buffer (Servicebio, Wuhan, China). The protein samples were denatured by boiling in water and then separated via SDS‐PAGE electrophoresis (Servicebio, Wuhan, China). The resolved proteins were transferred onto a PVDF membrane (Thermo Scientific, Waltham, USA). The membrane was blocked with 5% non‐fat milk in TBST solution (Servicebio, Wuhan, China) and incubated overnight at 4°C with primary antibodies against NLRP3 and β‐actin (Thermo Scientific, Waltham, USA). Following the incubation with primary antibodies, the membrane was incubated with the corresponding secondary antibody. Protein bands were visualized using a chemiluminescent imaging system (Thermo Scientific, Waltham, USA). The intensity of the bands was quantitatively analyzed using ImageJ software (National Institutes of Health, Bethesda, MD, USA). The experiment was independently conducted with three biological replicates.

### 2.12 Statistical Analysis

The data were processed and analyzed using Microsoft Excel and SPSS 20.0. Error bars represent the standard deviation (±SD) calculated from multiple biological replicates. Statistical significance was evaluated using Student's *t*‐test and Tukey's test. Significant differences were indicated as * or # (*p* < 0.05) and highly significant differences as ** or ## (*p* < 0.01). Specifically, # denoted a significant difference between the model group and the control group, while * denoted significant differences between the MP or Sil groups compared to the model group.

## 3 Results

### 3.1 Structural Characteristics of Morchella Mycelium Polysaccharides

The monosaccharide composition of the extracted *Morchella* polysaccharides was analyzed using HPLC, with the results illustrated in Figure S1a,b. By comparing the retention times of monosaccharide standards, it was determined that the polysaccharide isolated from *Morchella* primarily consists of glucose, glucuronic acid, ribose, galacturonic acid, rhamnose, and mannose. The molar ratios of these six monosaccharides were calculated to be 96.555:0.599:0.301:0.233:0.137:0.136, respectively. These findings indicated that glucose constitutes the predominant component of the monosaccharide composition, accounting for over 95%, thereby suggesting that glucose forms the backbone structure of the polysaccharide.

The relative molecular mass of the polysaccharide was measured using high‐performance gel permeation chromatography (HPGPC). The results showed a weight‐average molecular weight of 5.7 kDa with a polydispersity index (PDI) of 4.32. Nevertheless, the main peak contributed to 99% of the total population, indicating that the polysaccharide fulfilled the experimental requirements (Figure S1c).

Infrared spectroscopy was performed on the polysaccharide, and the resulting spectrum exhibited characteristic absorption peaks typical of polysaccharides (Figure S1d). The absorption band at 3382.11 cm^−1^ was assigned to O—H stretching vibrations, suggesting the presence of inter‐ or intramolecular hydrogen bonding, a common feature in polysaccharides. The bands at 2927.00 and 1368.87 cm^−1^ were attributed to C—H stretching and bending vibrations, respectively, confirming the existence of alkyl groups within the polysaccharide structure. A peak at 1651.94 cm^−1^ corresponded to C=O stretching vibrations, indicating the presence of functional groups such as uronic acid, which aligns with the monosaccharide composition analysis. The absorption at 1024.35 cm^−1^ was associated with C—O—C stretching vibrations, characteristic of glycosidic linkages in polysaccharides. Additionally, the band at 1153.12 cm^−1^, corresponding to C—O stretching vibrations, together with the peak at 1024.35 cm^−1^, reflected the skeletal vibrations of the pyranose ring. Finally, the absorption at 849.07 cm^−1^ was close to the characteristic frequency of *α*‐type glycosidic bonds, suggesting an *α*‐configuration in the polysaccharide.

### 3.2 Protective Effect of Morchella Mycelium Polysaccharides on the Mouse Liver

**FIGURE 1:**
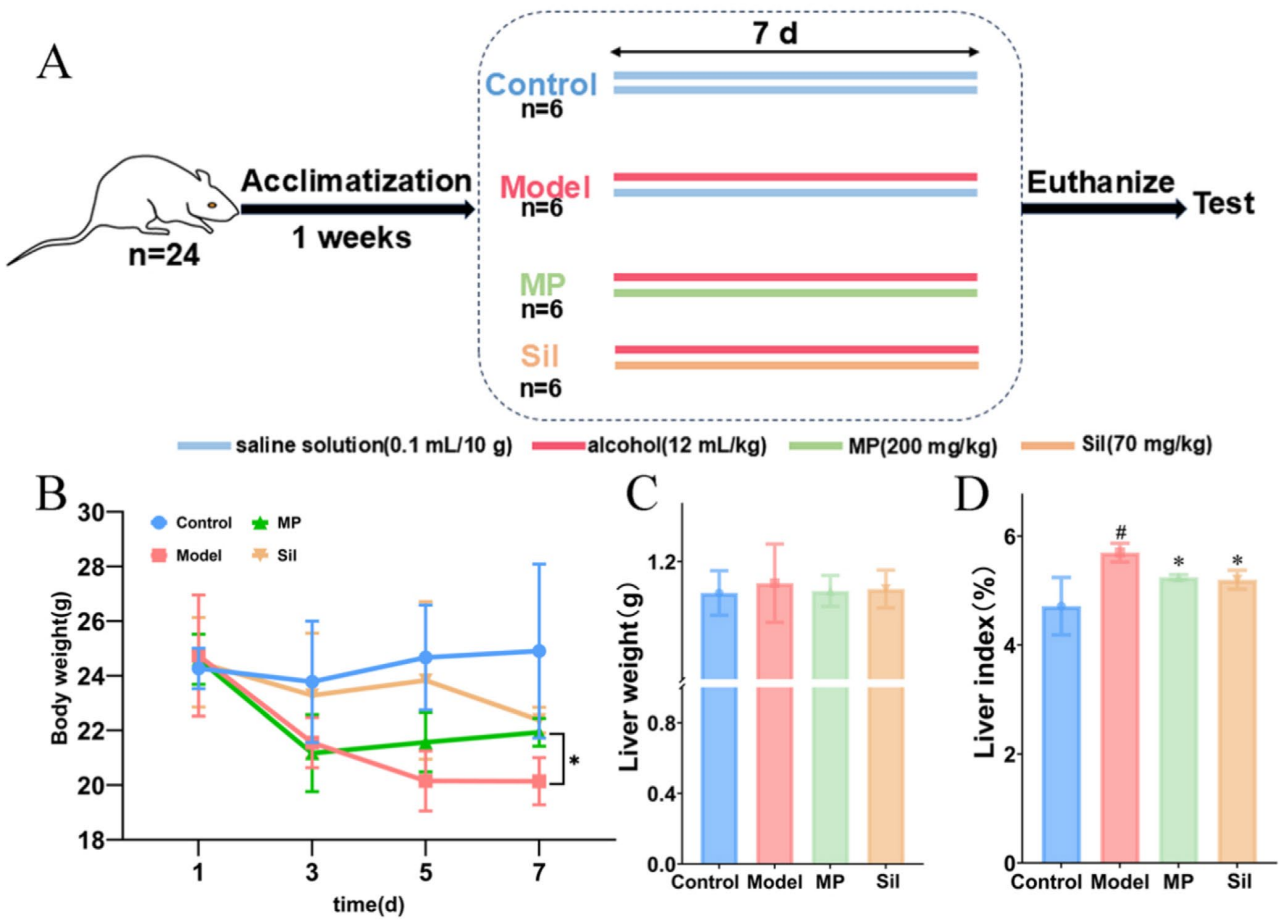
Establishment of a mouse model of alcoholic liver injury and analysis of the effects of *Morchella* polysaccharides on body weight and liver indices. (a) Experimental protocol for investigating the effects of *Morchella* polysaccharide on mice with alcoholic liver injury. (b) Impact of *Morchella* polysaccharide on body weight in ALI mice. (c) Influence of *Morchella* polysaccharide on liver weight in ALI mice. (d) Effects of *Morchella* polysaccharide on liver indices in ALI mice. # indicated significant difference (*p* < 0.05) between model and control groups; * indicated significant difference (*p* < 0.05) in MP or Sil group vs. Model group.

The body weight of the mice was measured and recorded every other day throughout the 7‐day modeling period (Figure 1a). A significant reduction in body weight was observed following alcohol exposure. Conversely, the liver weight in the model group was significantly higher than that in all other groups (Figure 1b,c). Based on the calculation of the liver index, it was evident that alcohol exposure induced a marked increase in the liver index. In contrast, the liver index in the mice treated with polysaccharides exhibited a downward trend relative to the model group (Figure 1d).

**FIGURE 2:**
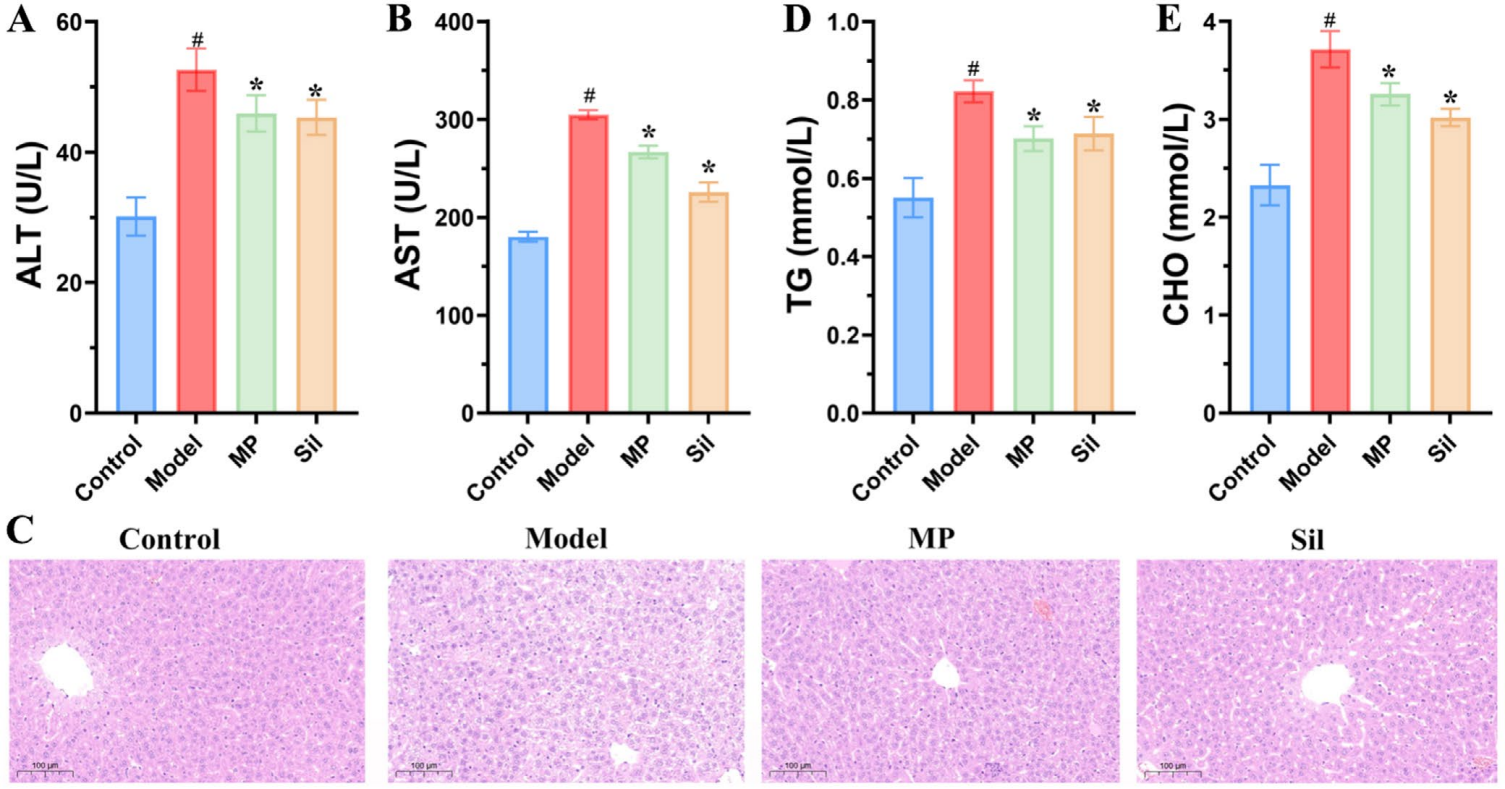
Effects of *Morchella* polysaccharide on serum liver enzyme levels, liver lipids, and pathological changes of liver tissues in mice with alcoholic liver injury. The enzymatic activities of ALT (a) and AST (b) in serum. (c) Hematoxylin–eosin staining. The concentrations of TC (d) and CHO (e). # indicated significant difference (*p* < 0.05) between model and control groups; * indicated significant difference (*p* < 0.05) in MP or Sil group vs. Model group.

AST and alanine ALT are commonly used as indicators for evaluating liver function or detecting liver damage. To confirm the successful establishment of an alcohol‐induced liver injury mouse model, we measured the serum levels of AST and ALT. The results demonstrated a significant elevation in serum AST and ALT concentrations in the model group compared to the control group (Figure 2a,b). Notably, the examination of liver histopathological sections revealed that the hepatocytes in the control group exhibited a well‐organized arrangement with round nuclei. Conversely, in the model group, liver cells exhibited a loose arrangement, increased volume, and signs of necrosis in some cells (Figure 2c). These results indicated that the ALI model was successfully established. Following the administration of MP, the serum levels of AST and ALT in mice were markedly reduced compared to the model group, although these levels remained higher than those in the control group (Figure 2a,b). Microscopic examination of the liver tissue following HE staining revealed a substantial reduction in hepatocellular damage in the MP‐treated group, with notable improvements in cellular loosening and enlargement, as well as a marked decrease in cell necrosis compared to the model group (Figure 2c). These indices were significantly lower in the silymarin‐treated group compared to the model group, with a notable reduction in liver tissue cell damage (Figure 2a–c). In lipid metabolism, the levels of TG and CHO serve as critical indicators of hepatic injury. Relative to the control group, the serum levels of TG and CHO were markedly elevated in alcohol‐exposed mice. However, compared to the model group, the MP resulted in a significant decrease in TG and CHO levels, demonstrating an effect comparable to that of silymarin (Figure 2d,e).

### 3.3 Levels of Oxidative Stress Markers and Inflammatory Factors in Mouse Liver Tissues

**FIGURE 3:**
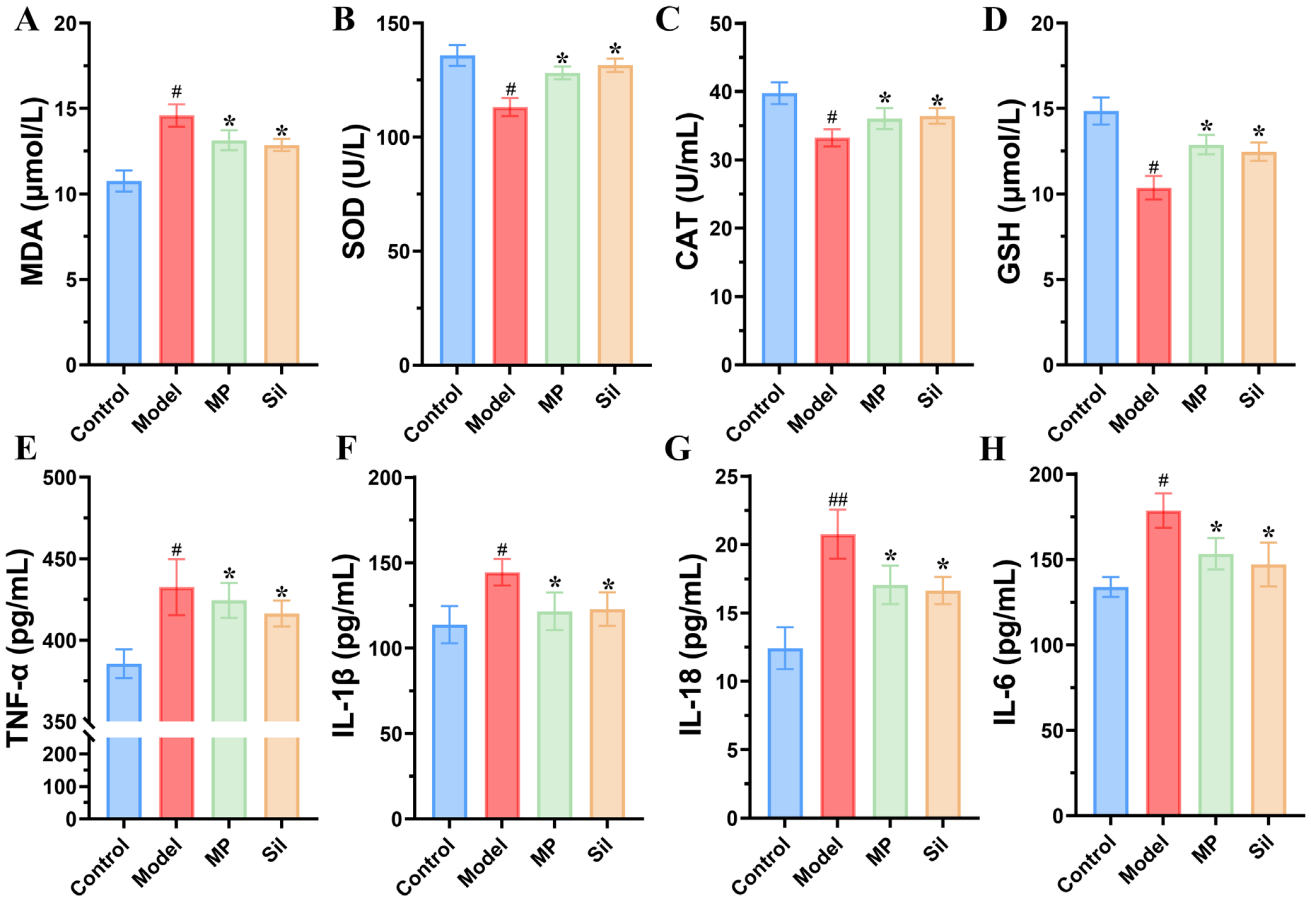
Effects of *Morchella* polysaccharides on the contents of oxidative stress markers and cytokines in the livers of ALI mice. (a) MDA contents. (b) SOD contents. (c) CAT contents. (d) GSH contents. (e) TNF‐α contents. (f) IL‐1β contents. (g) IL‐18 contents. (h) IL‐6 contents. # indicated significant difference (*p* < 0.05) between model and control groups; * indicated significant difference (*p* < 0.05) in MP or Sil group vs. Model group.

Excessive alcohol intake can induce oxidative stress in the liver, resulting in an overproduction of reactive oxygen species (ROS). Accumulation of ROS within cells leads to the oxidation of macromolecules, including proteins and DNA, culminating in hepatocyte apoptosis. To further investigate the protective effects of MP, we quantified the levels of oxidative markers in liver tissues (Figure 3a–d). Compared with the control group, the levels of MDA were markedly elevated in the liver tissues of alcohol‐fed mice, whereas the levels of SOD, CAT, and GSH were significantly reduced. Notably, the MDA levels in the MP‐treated group were significantly lower than those in the model group, with a reduction of approximately 10%. Additionally, the levels of antioxidant enzymes were significantly higher in the MP group compared to the model group, with SOD, CAT, and GSH increasing by 13.24%, 24.35%, and 8.44%, respectively.

Cytokines are key mediators of inflammatory responses and immune‐related liver damage. Pro‐inflammatory cytokines, such as TNF‐α, IL‐1β, and IL‐18, contribute to the exacerbation of inflammation and liver injury, whereas anti‐inflammatory cytokines, including IL‐10, IL‐6, and IL‐13, demonstrate protective effects and mitigate liver damage. Consequently, compared with the control group, the levels of TNF‐α, IL‐1β, IL‐18, and IL‐6 in the liver of the model group were significantly increased by 12.20%, 26.94%, 67.04%, and 25.01%, respectively. Following the administration of MP, the levels of TNF‐α, IL‐1β, IL‐18, and IL‐6 were markedly decreased by 2.90%, 15.83%, 17.84%, and 14.19%, respectively, compared with those in the model group (Figure 3e–h).

### 3.4 Transcriptomics Analysis of the Hepatoprotective Effects of Morchella Mycelium Polysaccharides

**FIGURE 4:**
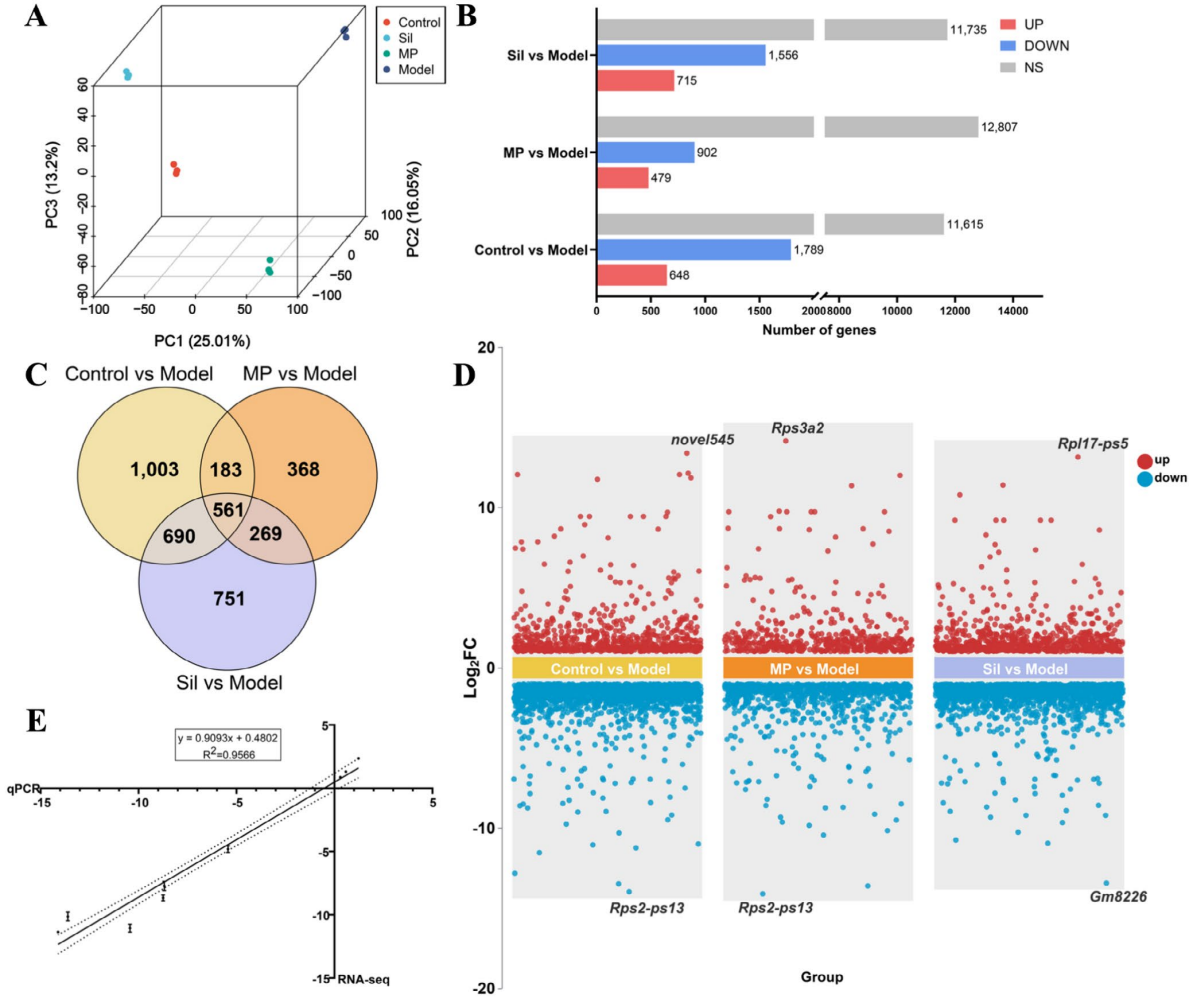
Overview of transcriptome data. (a) Results of principal component analysis. (b) Statistical outcomes of DEG screening. (c) Venn diagram of DEGs. (d) Volcano plot of DEGs. (e) Results of qPCR validation.

To further investigate the liver‐protective mechanism of MP, total RNA was extracted from liver tissue samples across four experimental groups for transcriptome sequencing. Transcriptome sequencing generated a total of 690 million raw reads, with an average of 57.35 million raw reads per sample (Table S2). After quality filtering, 680 million clean reads were obtained, averaging 56.6 million clean reads per sample. The Q30 value averaged higher than 97%, confirming high sequencing quality. Based on gene expression levels, principal component analysis (PCA) was performed on each sample, revealing that PC1 accounted for 25.01%, PC2 accounted for 16.05%, and PC3 accounted for 13.2% (Figure 4a). These results indicated distinct clustering among samples and excellent intra‐group repeatability. Through differential gene expression analysis, a total of 3825 differentially expressed genes (DEGs) were identified, of which 561 genes exhibited significant differences in expression across treatment groups (Figure 4b,c). Compared with the model group, the control group showed 1789 downregulated DEGs, with Rps2‐ps13 exhibiting the largest downregulation (log_2_ fold change = −13.98). Among the 648 upregulated DEGs, novel545 demonstrated the highest upregulation (log_2_ fold change = 13.39) (Figure 4b,d). In the MP group, compared with the model group, there were 1556 downregulated DEGs, with Gm8226 showing the largest downregulation (log_2_ fold change = −13.43). Additionally, 715 upregulated DEGs were identified, with Ppl17‐ps5 exhibiting the highest upregulation (log_2_ fold change = 13.15) (Figure 4b,d). Compared with the model group, a total of 902 down‐regulated DEGs were identified in the Sil group, among which Rps2‐ps13 exhibited the highest down‐regulation ratio, with a log_2_ fold change of −14.11. Furthermore, 479 upregulated DEGs were detected, with Rps3a2 demonstrating the most significant upregulation (log_2_ fold change = 14.16) (Figure 4b,d). To validate the transcriptome sequencing results, we randomly selected five genes for qPCR analysis. Pearson correlation analysis revealed a significant positive correlation between the qPCR results and the transcriptome sequencing data (*R* ^2^ = 0.9566), confirming the reliability of the sequencing results (Figure 4e).

To elucidate the functions of the DEGs, we performed GO functional enrichment analysis and KEGG metabolic pathway enrichment analysis. The results of the GO functional enrichment analysis are presented in Figure S2. Compared with the model group, the functions of DEGs in the control group were primarily associated with the cytoplasm, membrane, protein binding, cell periphery, and positive regulation of biological processes. In the Sil group, the functions of DEGs were predominantly concentrated in intracellular anatomical structures, binding activities, cytoplasmic localization, protein binding, and ion binding. In the MP group, the functions of DEGs were mainly focused on binding activities, cytoplasmic localization, membrane association, and protein binding.

**FIGURE 5:**
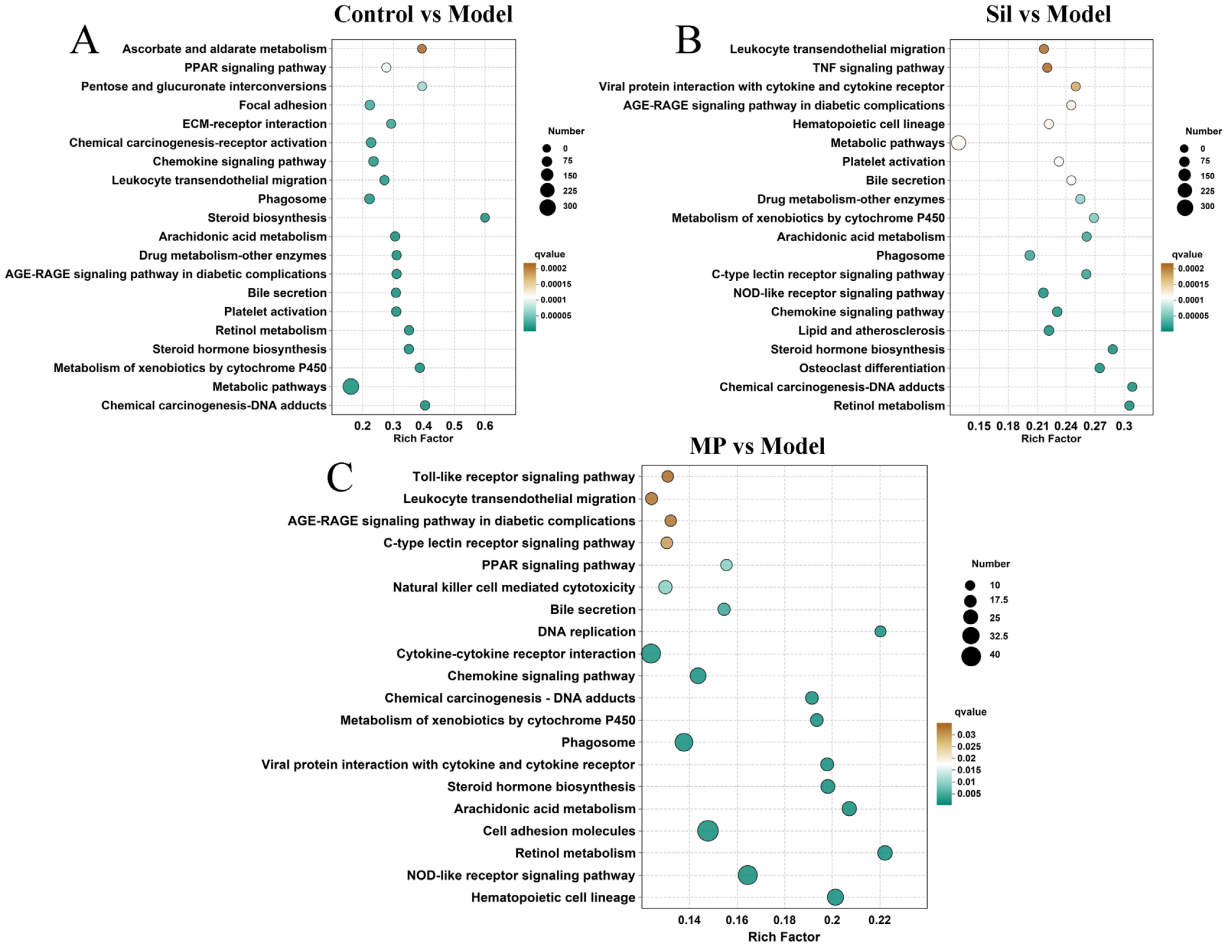
KEGG enrichment analysis results of DEGs. (a) Control vs. Model; (b) Sil vs. Model; and (c) MP vs. Model.

The results of the KEGG metabolic pathway enrichment analysis are presented in Figure 5. Compared with the model group, DEGs in the control group were primarily enriched in pathways such as chemical carcinogens‐DNA adducts, xenobiotic metabolism by cytochrome P450, and steroid hormone biosynthesis. Within the Sil group, DEGs were mainly associated with pathways including retinol metabolism, chemical carcinogens‐dna adducts, and osteoclast differentiation. For the MP group, DEGs were largely concentrated in the NOD‐like receptor signaling pathway, cell adhesion molecules, and cytokine‐cytokine receptor interaction.

### 3.5 Analysis of the Protective Effects of Morchella Polysaccharides via Weighted Gene Co‐Expression Network Analysis

**FIGURE 6:**
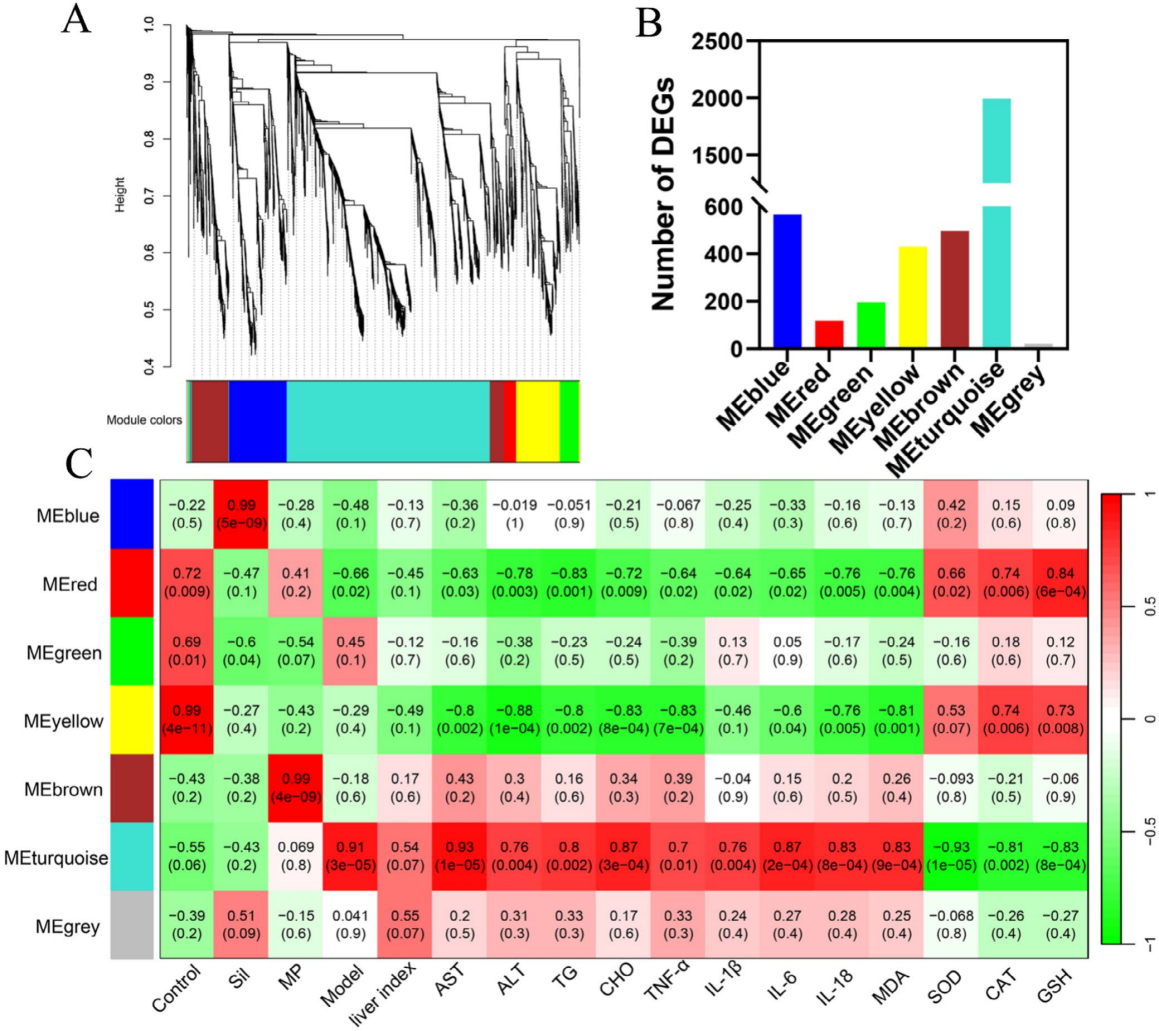
WGCNA analysis of DEGs. (a) Dendrogram illustrating the aggregation of distinct genes into co‐expression modules. (b) Count of differentially expressed genes within various co‐expression modules. (c) Association between co‐expression modules and traits.

To elucidate the co‐expression modules of the control group, model group, Sil group, and MP group, a co‐expression network was constructed using WGCNA based on 3825 DEGs (Figure 6a). A total of seven distinct co‐expression modules were identified, comprising 566, 119, 196, 431, 497, 1994, and 22 DEGs in the blue, red, green, yellow, brown, turquoise, and gray modules, respectively (Figure 6b). Furthermore, the relationships between these modules and phenotypes were systematically analyzed using WGCNA (Figure 6c). Notably, the yellow module exhibited the highest positive correlation with the control group (*r* = 0.99). The blue module showed the strongest positive correlation with the Sil group (*r* = 0.99), suggesting that the genes within this module were closely associated with the treatment of ALI in the Sil group. The brown module demonstrated the highest positive correlation with the MP group (*r* = 0.99), indicating that its genes were strongly related to the hepatoprotective effects of MP. The turquoise module had the strongest positive correlation with the model group (*r* = 0.91), implying that its genes were closely linked to ALI in mice. Importantly, the turquoise module also exhibited significant positive correlations with AST, CHO, IL‐1β, IL‐6, IL‐18, and MDA, with r values of 0.93, 0.87, 0.76, 0.87, 0.83, and 0.83, respectively. Additionally, it displayed significant negative correlations with SOD, CAT, and GSH, with *r* values of −0.93, −0.81, and −0.83, respectively, further validating the close relationship between these physiological and biochemical indices and ALI.

GO function and KEGG metabolic pathway enrichment analyses were performed for the genes associated with each module (Figures S3 and S4). In the blue module, the functions of DEGs were primarily enriched in enzyme binding and iron ion binding, among others. These genes exhibited significant associations with metabolic pathways such as retinal metabolism, steroid hormone biosynthesis, and lipid and atherosclerosis metabolism. In the red module, the functions of DEGs were predominantly enriched in early endosome localization and cholesterol biosynthetic processes. The major metabolic pathways enriched in this module included phagosome, bile secretion, metabolism of xenobiotics by cytochrome P450, and terpenoid backbone biosynthesis. For the green module, the functions of DEGs were mainly related to GTP binding, GTPase activity, and cellular response to interferon‐gamma. These genes demonstrated significant correlations with hematopoietic cell lineage, Epstein–Barr virus infection, and other metabolic pathways. In the yellow module, the functions of DEGs were primarily enriched in enzyme binding, iron ion binding, oxidoreductase activity, and heme binding. The significantly enriched metabolic pathways included steroid hormone biosynthesis, retinol metabolism, and chemical carcinogen‐receptor activation. In the brown module, the functions of DEGs were mainly associated with protease binding, heparin binding, and heme binding. The primary enriched metabolic pathways were the PPAR signaling pathway, fatty acid degradation, and cell adhesion molecules. The functions of DEGs in the turquoise module were primarily associated with inflammatory response, cytoskeletal organization, and actin binding. The major enriched metabolic pathways included the NOD‐like receptor signaling pathway, chemokine signaling pathway, and lipid metabolism‐related pathways involved in atherosclerosis. In the gray module, most DEGs were functionally concentrated in ciliary basal body organization, protein serine kinase activity, intracellular signal transduction, protein serine/threonine/tyrosine kinase activity, and axonal processes. Additionally, the predominant metabolic pathways enriched in this module included the IL‐17 signaling pathway and DNA mismatch repair mechanisms, among others.

### 3.6 Role of the NOD‐Like Receptor Signaling Pathway in Mediating the Hepatoprotective Effects of Morchella Polysaccharides

**FIGURE 7:**
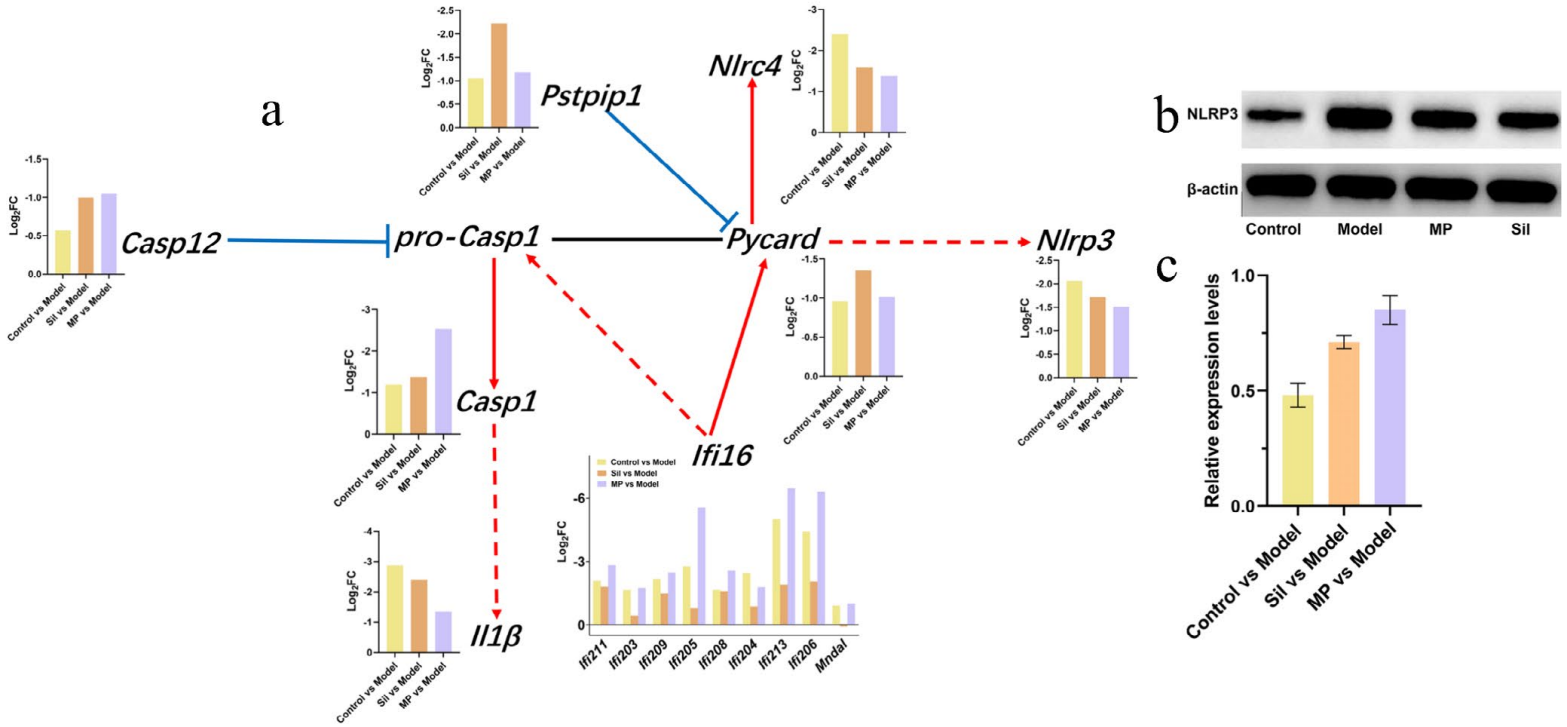
Regulatory mechanisms of the NOD‐like receptor signaling pathway. (a) Expression levels of key genes in the NOD‐like receptor signaling pathway. (b) Western blot analysis of NLRP3 protein expression. (c) Quantitative analysis of NLRP3 protein expression levels.

By systematically analyzing the enrichment of DEGs in metabolic pathways, we identified that the NOD‐like receptor signaling pathway was associated with the hepatoprotective effect of MPs. Further investigation into the expression levels of DEGs within this pathway revealed that, compared to the model group, most genes involved in this metabolic pathway were down‐regulated following MP treatment, with the exception of *Camp* (Figure S5 and Figure 7a). Through an integrated analysis of gene regulation in the NOD‐like receptor signaling pathway and transcriptome‐detected gene expression (Figure 7a), we observed that *Casp12* inhibited *pro‐Casp1* expression, while *Ifi16* promoted its expression. Given the substantial fold‐change differences in *Ifi16* expression, the overall down‐regulation of *pro‐Casp1* resulted in a significant reduction in *Casp1* expression. Additionally, *Ifi16* also contributed to the down‐regulation of *Pycard*. Since *Pycard* can regulate the expression of *Nlrp3*, the down‐regulation of *Pycard* led to a corresponding decrease in *Nlrp3* expression. To validate the down‐regulation of *Nlrp3*, we conducted Western blot analysis on liver tissue samples. The results demonstrated that Nlrp3 expression was highest in the model group and lowest in the control group. Following MP treatment, Nlrp3 expression was significantly reduced (Figure 7b). Quantitative analysis of protein band grayscale further confirmed that MP intervention led to decreased Nlrp3 expression compared to the model group (Figure 7c).

## 4 Discussion

### 4.1 *Morchella* Has Garnered Significant Attention as a Highly Prized Edible Fungus due to Its Unique Flavor and Substantial Health Benefits

Its biological activities are primarily attributed to bioactive compounds such as polysaccharides, polyphenols, and proteins. The structural characteristics of polysaccharides serve as the foundation for their biological activity, with monosaccharide components influencing functional groups, spatial conformation, and other structural features. In this study, we systematically analyzed the monosaccharide composition of MPs and identified glucose as the predominant monosaccharide, which is consistent with previous findings (Cai et al. 2018; Kuang et al. 2022). Teng et al. reported that in *Morchella* polysaccharides, glucose accounted for 90.971%, galactose (Gal) for 0.161%, mannose (Man) for 0.145%, and glucuronic acid (GLC‐UA) for 0.094% (Teng et al. 2023). Chen et al. (2025) extracted polysaccharides primarily composed of D‐mannose, D‐glucose, and D‐galactose, with a molar ratio of 1.00:12.89:0.29. Among the top four monosaccharides—glucose, glucuronic acid, ribose, and galacturonic acid—glucose constituted up to 96.555%. Variations in monosaccharide composition may stem from differences in strains, culture conditions, or extraction methods (Chen et al. 2025). The high glucose content in these polysaccharides often correlates with β‐glucan structures, which are renowned for their potent immunomodulatory and bioactive properties. This suggests that the polysaccharides we extracted may exhibit biological activities comparable to those of β‐glucan‐rich fungal polysaccharides, such as those found in *Ganoderma lucidum* (Zhong et al. 2024) and *Lentinula edodes* (Dai et al. 2023). Studies have demonstrated that glucose derived from polysaccharides exhibits anti‐inflammatory (Lee et al. 2020), antioxidant (Lo et al. 2011), anti‐tumor (Liao et al. 2020), antibacterial (Hashemifesharaki et al. 2020), and prebiotic activities (Chen et al. 2019). Additionally, glucuronic acid demonstrates prebiotic activity (Yu et al. 2021), while galacturonic acid possesses both antioxidant (Yi et al. 2022) and prebiotic properties (Dou et al. 2021).

### 4.2 Weight‐Average Molecular Weight (Mw) of 5.7 kDa Observed in This Study Falls Within the Low‐Molecular‐Weight (LMW) Range (1–10 kDa) Characteristic of Fungal Polysaccharides

LMW polysaccharides are increasingly recognized to have enhanced bioavailability and bioactivity compared to high‐molecular‐weight (HMW) polysaccharides (Fan et al. 2025). Yuan et al. (2015) found that LMW polysaccharides isolated from *Morus alba* L. exhibited significantly higher in vitro antioxidant activity than their HMW counterparts. Extensive studies on gut microbiota have consistently shown that LMW polysaccharides are more effective in modulating the composition and function of intestinal microbiota than HMW polysaccharides (Fu et al. 2021; Wan et al. 2024). Additionally, LMW polysaccharides exhibit superior capacity in regulating blood glucose levels (Tong et al. 2023). The enhanced bioactivity of LMW polysaccharides stems from structural and physicochemical advantages: increased availability of free hydroxyl groups, greater exposure of bioactive sites, higher water solubility, and reduced viscosity. These properties collectively strengthen their interaction with biological systems. In contrast, the polysaccharides isolated from *Morchella* in the study by Teng et al. (Teng et al. 2023) were characterized as HMW compounds, highlighting a notable divergence from our findings. This discrepancy may be attributed to variations in the raw materials and extraction methodologies employed. Nevertheless, the polysaccharides identified in our study share an α‐configuration with those reported by Teng et al. Teng et al. (2023). Collectively, the α‐configuration, low molecular weight, and high glucose content of our isolated MPs suggest they may exert distinct biological effects via unique mechanistic pathways. Consequently, the aim of this study was to investigate the protective effects of MPs on ALI and elucidate the underlying mechanisms.

ALI is characterized by elevated levels of AST and ALT in the blood, which are critical biochemical markers for diagnosing such injuries (Zhang et al. 2023). In our study, alcohol exposure significantly increased AST and ALT levels in the blood of mice. However, intervention with the MP effectively attenuated these elevations in serum AST and ALT levels. Alcohol metabolism is intricately linked to lipid metabolism. Chronic excessive alcohol consumption can result in hepatic metabolic disturbances, including lipid accumulation in liver cells and dysregulation of lipid metabolism, such as enhanced triglyceride and fatty acid synthesis, along with reduced secretion of very low‐density lipoproteins (VLDL) (Zhang et al. 2018). Consistent with previous studies, the serum levels of TG and CHO were elevated in the alcohol‐treated group. Notably, treatment with the MP significantly decreased these levels, suggesting that the MP plays a crucial role in regulating lipid metabolism. This regulatory effect may be mediated through the inhibition of lipid synthesis or the promotion of fatty acid oxidation. A similar mechanism has been reported for cordycepin, which suppresses hepatic steatosis by activating the AMP‐activated protein kinase (AMPK) signaling pathway (Chen et al. 2022).

High alcohol consumption triggers oxidative stress in the liver, resulting in excessive production of ROS. Intracellular ROS accumulation causes the oxidation of macromolecules such as proteins and DNA, ultimately causing hepatocyte apoptosis (Salete‐Granado et al. 2023). However, liver cells possess a robust antioxidant defense system, including enzymatic antioxidants like SOD and catalase, as well as non‐enzymatic antioxidants such as GSH and ubiquitin (Allameh et al. 2023). Consequently, the activities of catalase and SOD, along with the levels of MDA and GSH, are frequently utilized as critical indicators for assessing cellular oxidative damage. In our study, following alcohol exposure, MDA content in the livers of mice increased, whereas the levels of SOD, catalase, and GSH decreased. MP treatment reduced MDA levels and enhanced antioxidant system indices, findings that align with previous studies (Xiong et al. 2016). Specifically, the MP alleviates oxidative damage in mouse liver tissues by scavenging ROS, reducing MDA content, and increasing antioxidant enzyme activity (Xiong et al. 2016). This mechanism shares similarities with the mode of action of exercise, as it combats ethanol‐induced reactive oxygen species generation through the activation of the Nrf2/Keap1‐HO‐1 pathway (Fathi et al. 2020).

Inflammation is a critical factor in the progression of ALI to steatohepatitis, cirrhosis, and HCC (Gao et al. 2019). Activated M1 macrophages release significant amounts of cytokines, including IL‐1β, TNF‐α, IL‐12, IL‐18, and IL‐23, which facilitate the induction of an inflammatory response in antigen‐specific Th1 and Th17 cells, thereby promoting inflammation (Gao et al. 2019). Certain cytokines, such as TNF‐α and IL‐6, exhibit dual roles in the pathogenesis of ALI, contributing not only to inflammation and tissue damage but also to liver regeneration (Szabo 2017). In our study, alcohol exposure significantly elevated the levels of inflammatory factors in mouse hepatocytes, whereas MP treatment markedly reduced these levels. This indicated that the MP effectively suppresses the expression of pro‐inflammatory factors. The findings by Teng et al. further confirm that the *Morchella* polysaccharide reduces the levels of IFN‐α, IFN‐γ, TNF‐α, IL‐6, and IL‐17, effectively inhibiting the expression of inflammatory mediators (Teng et al. 2023). In the AIL model, the liver tissue exhibits structural disruption and infiltration of inflammatory cells (Fan et al. 2022). Histological analysis revealed that the polysaccharide significantly alleviated hepatocyte necrosis and mitigated the inflammatory response, further validating its anti‐inflammatory properties. This anti‐inflammatory effect may be attributed to the suppression of pro‐inflammatory cytokines such as TNF‐α and IL‐6, which aligns with the mechanism of action of β‐glucan from *Lentinus edodes* (Mizuno and Minato 2024).

Through transcriptome sequencing, we identified 3825 DEGs, indicating that the gene expression profile of mouse liver tissues was significantly altered following the intervention with MP. In both the control group and the Sil group, the most downregulated gene was *Rps2‐ps13* (log_2_FC ≤ −13.98), which encodes a ribosomal protein pseudogene. This pseudogene is closely associated with translation regulation and stress response mechanisms. The pronounced suppression of *Rps2‐ps13* in liver injury models may reflect a conserved adaptive mechanism initiated by the liver under stress, potentially reducing the demand for protein synthesis. This phenomenon has been previously documented to enhance the survival of liver cells under oxidative damage conditions (Chen et al. 2023). Conversely, the MP‐specific upregulated gene was *Ppl17‐ps5* (log_2_FC = 13.15), a pseudogene presumed to function as a protease inhibitor. The upregulation of *Ppl17‐ps5* indicates that the MP may exert a protective effect by promoting the turnover of protective proteins or inhibiting excessive proteolysis. This finding is consistent with previous reports regarding the regulation of extracellular matrix remodeling during liver fibrosis (Ortiz et al. 2021).

Enrichment analysis of DEGs revealed significant enrichment in the NOD‐like receptor signaling pathway under the intervention of MPs. WGCNA analysis further demonstrated that the metabolic pathway was associated with the alcohol treatment group. Zhang et al. (2025) also identified a strong correlation between the NOD‐like receptor signaling pathway and ALD through transcriptome sequencing. Activation of the NOD‐like receptor signaling pathway, particularly the NLRP3 inflammasome, is a key mediator of alcohol‐induced hepatitis and liver fibrosis (Wang et al. 2020; Gong et al. 2016). Transcriptomic data indicated that MP treatment significantly downregulated most genes in the NLR signaling pathway (except *Camp*) compared to the model group, suggesting that the MP effectively suppresses NRL‐mediated inflammatory cascades. Further investigation revealed that the regulation is mediated by differential modulation of *pro‐Casp1* expression by *Casp12* and *Ifi16*: *Casp12* inhibits *pro‐Casp1* expression, whereas *Ifi16* promotes its transcription. Notably, the transcriptional fold change of *Ifi16* was substantial (log_2_FC > −4.5), leading to reduced net expression of *pro‐Casp1* and consequently diminished production of active *Casp1*. Given that activated *Casp1* is a pivotal driver of inflammasome maturation and pyroptosis, these processes are closely linked to liver injury (Aki et al. 2022). Therefore, the inhibitory effect of the MP on *Casp1* activation holds great promise for alleviating liver inflammation. At the same time, the down‐regulation of *Pycard* expression mediated by *Ifi16* further corroborates the suppression of NLR signaling. *Pycard* serves as a critical adaptor protein in the assembly of the NLRP3 inflammasome, and its transcriptional level is markedly inhibited by the MP, which may represent a key mechanism underlying the reduction in both NLRP3 mRNA and protein levels. Western blot analysis demonstrated that MP intervention significantly attenuated the expression level of NLRP3 proteins in the liver compared to the model group, with quantitative analysis revealing a reduction in band intensity by approximately 40%. These findings were highly consistent with transcriptomic data. These observations align with the existing literature, indicating that medicinal fungal polysaccharides exert anti‐inflammatory effects through the inhibition of NLRP3 inflammasome activation, thereby effectively mitigating pyroptosis and fibrosis in hepatocytes (Yu et al. 2023).

The observed downregulation of the NLR signaling axis—from diminished Ifi16‐Pycard interaction to enhanced NLRP3 suppression—suggests that the MP may interfere with the initiation and assembly of inflammasome activation. In the ALD mouse model, the NLRP3 inflammasome is activated, accompanied by significant elevation in the expression levels of *ASC*, *Casp1*, and *IL‐1β*. Notably, increased expression of inflammasome components (*IL‐1β*, *IL‐18*, *Casp1*) has been documented in patients with alcoholic liver disease and is associated with hepatic injury (Wu et al. 2017). In addition, antimicrobial peptides (Camp), as host defense peptides with well‐documented anti‐inflammatory properties, may exert a compensatory function in maintaining mucosal barrier integrity when the NLR pathway is inhibited. In this study, following MP treatment, *Camp* did not reduce expression in parallel with other genes within the pathway, but maintained a distinct expression pattern. This observation suggests that MP‐mediated regulation of the NOD‐like receptor signaling pathway is not characterized by uniform suppression, but rather involves the precise modulation of the gene expression network to achieve a hepatoprotective effect. From the perspective of pathway regulatory logic, the *Ifi16*‐mediated down‐regulation of core inflammatory genes—including *pro‐Casp1*, *Pycard*, and *Nlrp3*—contrasts with the sustained expression of *Camp*, thereby establishing a regulatory equilibrium defined by the coordinated suppression of pro‐inflammatory signaling and preservation of antimicrobial peptide production. This differential regulation likely enables the MP to maintain *Camp* expression while attenuating *Nlrp3* activation, resulting in a synergistic protective effect: on one hand, downregulation of *Nlrp3* limits excessive inflammation and prevents collateral tissue damage; on the other hand, preserved *Camp* expression enhances microbial clearance by targeting translocated pathogens in the liver. By reducing the persistent activation of NOD‐like receptor pathways via pathogen‐associated molecular patterns such as lipopolysaccharide, this dual mechanism mitigates chronic inflammation and reinforces innate immunity—forming an integrated hepatoprotective strategy that balances immune modulation and antimicrobial efficacy. Previous studies have demonstrated that Camp plays a regulatory role in maintaining microbial homeostasis in ALD, and its deficiency is linked to heightened hepatic pro‐inflammatory responses to alcohol, elevated oxidative stress levels, and more severe intestinal inflammation in a murine model (Hu et al. 2019; Li et al. 2020).

This dual regulatory mechanism—suppressing pro‐inflammatory NLR components while enhancing protective antimicrobial peptides—further elucidates the pleiotropic regulation of the MP on hepatic immune homeostasis. Notably, while the polysaccharide demonstrated significant efficacy in partially restoring liver function, its incomplete reversal of alcohol‐induced damage suggests potential dose limitations or involvement of other mechanisms. Future studies should focus on structural characterization (e.g., methylation/NMR analysis), synergistic mechanisms (e.g., regulation of the gut‐liver axis), or leverage third‐generation sequencing technologies for in‐depth investigations into novel genes and alternative splicing at the transcriptome level (Wu et al. 2017; Zhang et al. 2024).

## 5 Conclusions

The mycelial polysaccharide extracted from *Morchella* was primarily composed of glucose monosaccharides and exhibited significant multi‐target protective effects against ALI. The key manifestations included the reduction of serum ALT and AST levels, improvement in lipid metabolism (as evidenced by decreased triglyceride and cholesterol content), and restoration of the histopathological structure of hepatocytes. Mechanistic investigations revealed that the polysaccharide alleviates oxidative stress by enhancing the activity of antioxidant enzymes (e.g., superoxide dismutase, catalase, and glutathione peroxidase) and inhibiting lipid peroxidation (as indicated by reduced malondialdehyde levels). Transcriptomic analysis further demonstrated that the polysaccharide downregulates critical regulatory nodes such as *Ifi16*, *Pycard*, and *Nlrp3* by suppressing the Nlrp3 inflammasome within the NOD‐like receptor signaling pathway, thereby inhibiting *pro‐Casp1* activation and subsequent pyroptosis of hepatocytes. Additionally, the polysaccharide upregulated the expression of antimicrobial peptides (*Camp*), highlighting its dual mechanism of anti‐inflammatory action and barrier protection. This study enhances our understanding of the *Morchella* polysaccharide as a highly effective natural compound for combating ALI and elucidates the relationship between its structural specificity and multi‐target bioactivity.

## Author Contributions

Conceptualization: X.H., Q.D., Y.Z., and Z.W.; methodology: X.H., Q.D., L.M., and T.Z.; software: X.H. and T.Z.; validation: X.H., Q.D., L.M., M.Z., Z.C., W.L., and K.H.; formal analysis: X.H., Q.D., M.Z., Z.C., W.L., and K.H.; investigation: X.H., L.M., T.Z., M.Z., Z.C., W.L., and K.H.; resources: Y.Z. and Z.W.; data curation: X.H., Q.D., and T.Z.; writing – original draft preparation: X.H., Q.D., and L.M.; writing – review and editing: X.H., Q.D., and Y.M.; visualization: X.H. and T.Z.; supervision: X.H., Q.D., Y.Z., and Z.W.; project administration: X.H., Q.D., and Z.W.; and funding acquisition: X.H., Q.D., and Z.W.

## Funding

The research was financially supported by the Zhumadian biomedical industry research and development joint fund project (CY2523023), the Science and Technology Development Plan Project of Henan Province, China (232102310338), the Key scientific research project of Henan Higher Education Institutions (24B550006), the National Scientific Research Project Cultivation Fund project of Huanghuai University (XKPY‐2023001), the Henan Province Edible Fungi Industry Technology System Xinyang Comprehensive Experiment Station (HARS‐22‐08‐Z3), and the Science and Technology Development Plan Project of Henan Province, China (232102110230).

## Disclosure

Institutional review board statement: The animal study was reviewed and endorsed by the Experimental Animal Ethics Committee of Huanghuai University (Approval No. HHU20220008), approved on 20 September 2022.

## Conflicts of Interest

The authors declare no conflicts of interest.

## Supporting information


**Figure S1:** Structural Characteristics of the *Morchella* mycelium polysaccharide. (a) Test results for monosaccharide standards. (b) Test results for polysaccharide samples. (c) Relative molecular mass of polysaccharide samples. (d) Infrared spectra of polysaccharide samples.

**Figure S2:** GO functional enrichment analysis of DEGs. (a) Control vs. Model, (b) Sil vs. Model, and (c) MP vs. Model.

**Figure S3:** GO analysis of DEGs in each co‐expression module. (a) Blue module; (b) red module; (c) green module; (d) yellow module; (e) brown module; (f) turquoise module; and (g) gray module.

**Figure S4:** KEGG analysis of DEGs in each co‐expression module. (a) Blue module; (b) red module; (c) green module; (d) yellow module; (e) brown module; (f) turquoise module; (g) gray module.

**Figure S5:** Expression of genes related to the NOD‐like receptor signaling pathway.

**Table S1:** List of primers used in this study.

**Table S2:** Overview of transcriptome data.

## Data Availability

The data that support the findings of this study are openly available in NCBI at https://dataview.ncbi.nlm.nih.gov/object/PRJNA1265903?reviewer=ppbm8p0d4h2mqde7669mpadfdp, reference number PRJNA1265903.
