# Supplementary figures and images for: Hepatoprotective Effect of Morchella Mycelia Polysaccharides on Alcoholic Liver Injury and Its Mechanism Involving the Modulation of NOD‐Like Receptor Signaling Pathway
Source: Food Sci Nutr. 2026 Jan 23;14(1):e71483. doi: 10.1002/fsn3.71483 (PMC12828981; doi:10.1002/fsn3.71483)

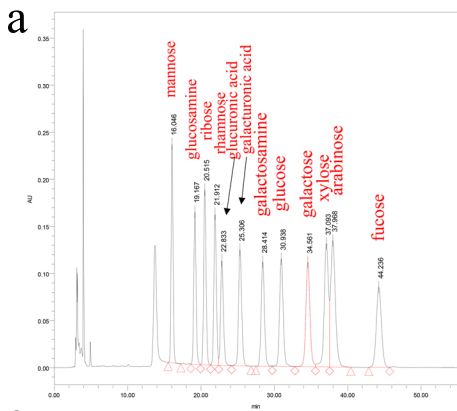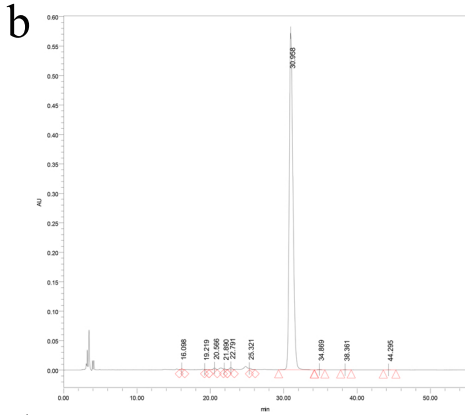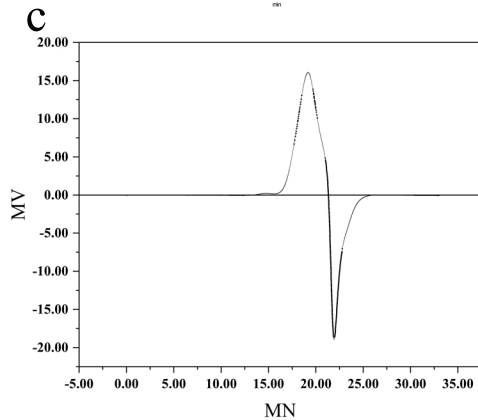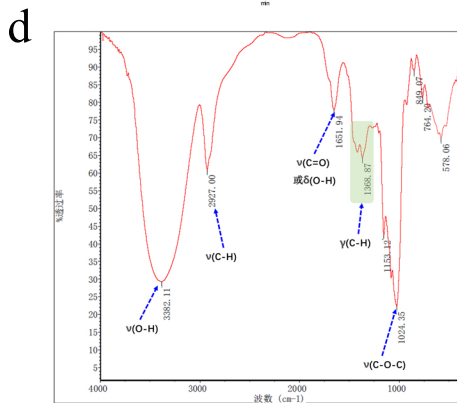

Supplement: Supplementary file 1 — Figure S1: Structural Characteristics of the Morchella mycelium polysaccharide. (a) Test results for monosaccharide standards. (b) Test results for polysaccharide samples. (c) Relative molecular mass of polysaccharide samples. (d) Infrared spectra of polysaccharide samples. [file FSN3-14-e71483-s005.pdf]

## Control vs Model

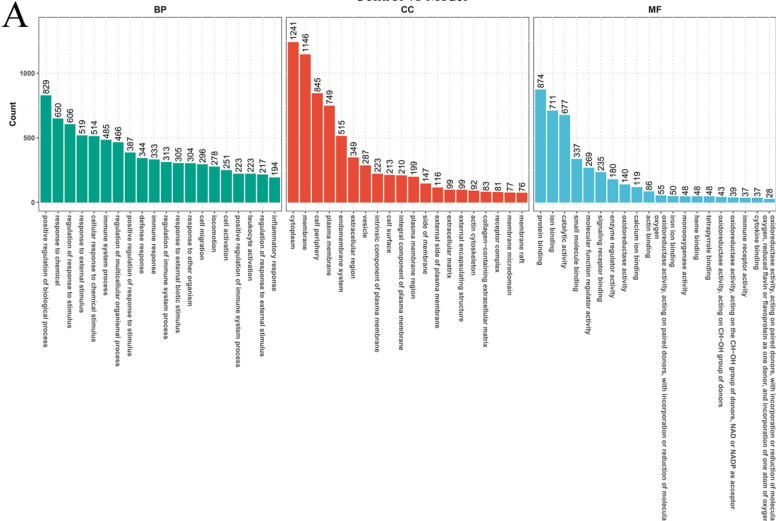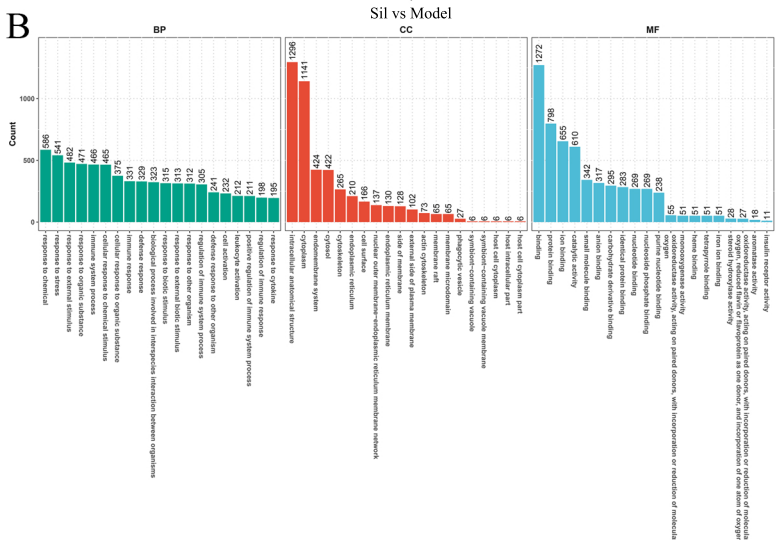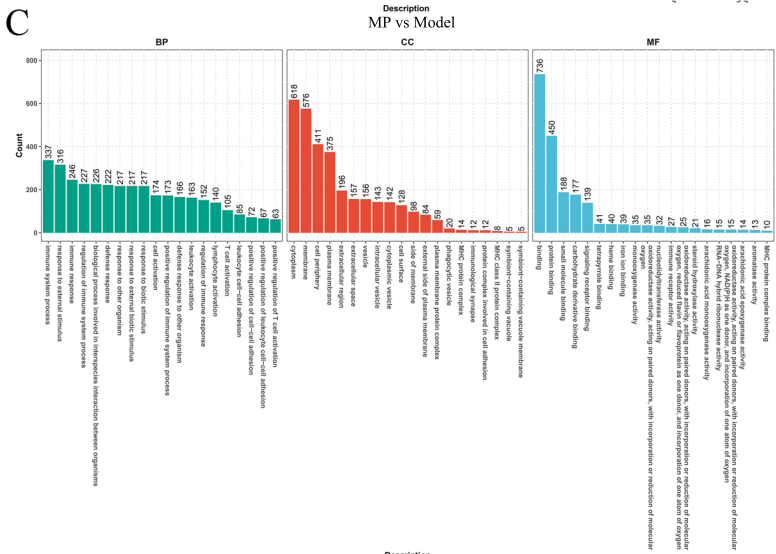

Supplement: Supplementary file 2 — Figure S2: GO functional enrichment analysis of DEGs. (a) Control vs. Model, (b) Sil vs. Model, and (c) MP vs. Model. [file FSN3-14-e71483-s006.pdf]

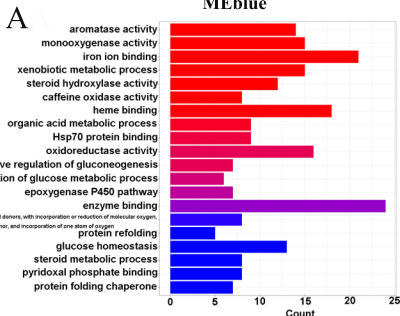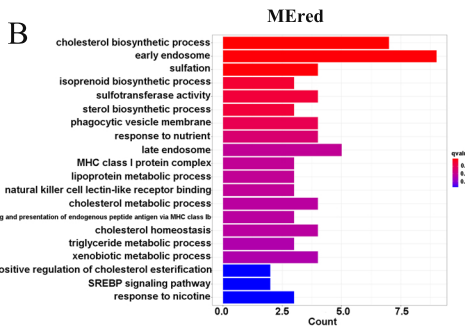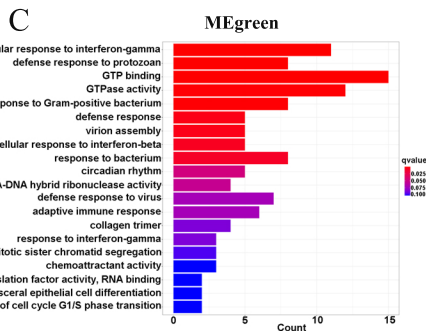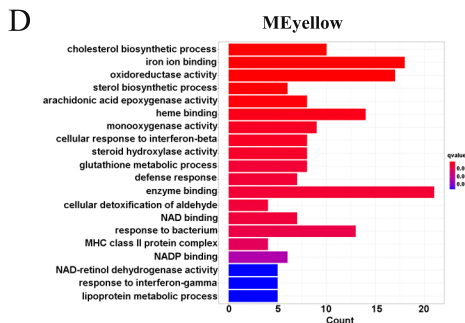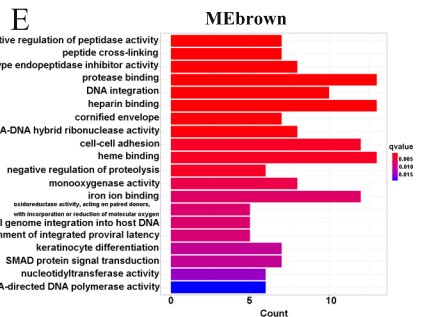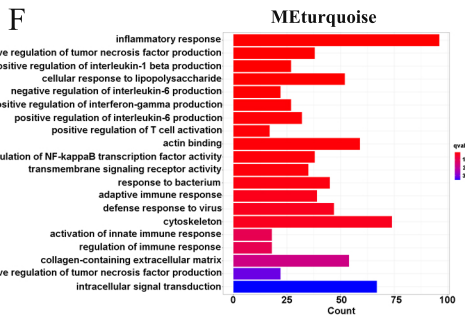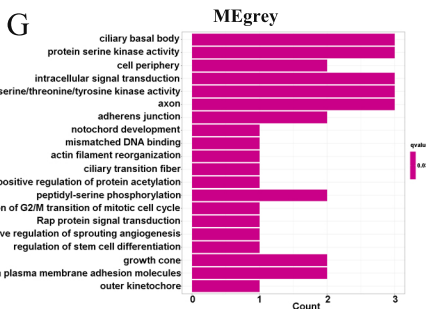

Supplement: Supplementary file 3 — Figure S3: GO analysis of DEGs in each co‐expression module. (a) Blue module; (b) red module; (c) green module; (d) yellow module; (e) brown module; (f) turquoise module; and (g) gray module. [file FSN3-14-e71483-s003.pdf]

A

MEblue

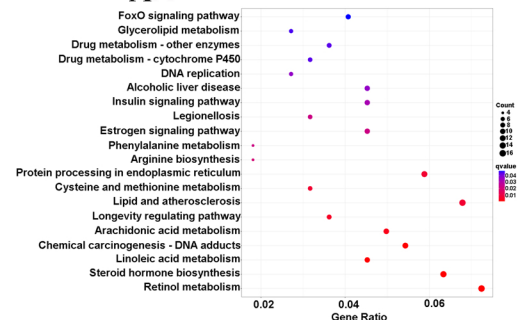

B

MERed

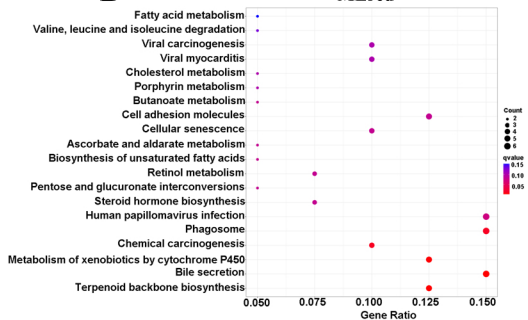

C

MEgreen

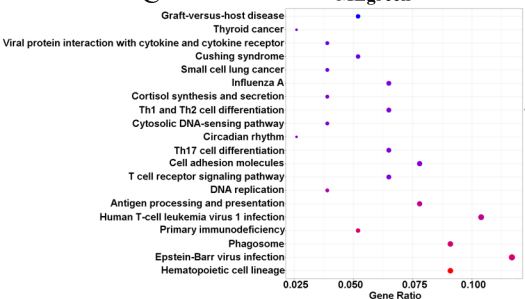

D

MEyellow

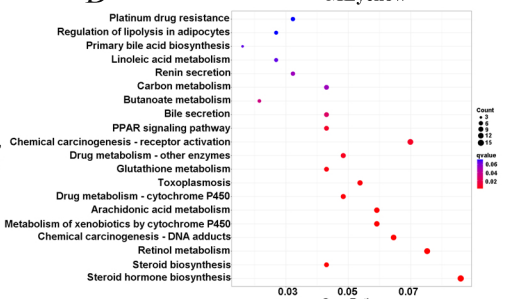

E

MEbrown

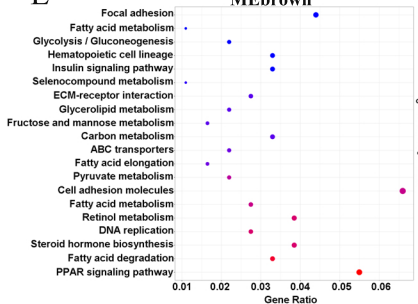

F

MEturquoise

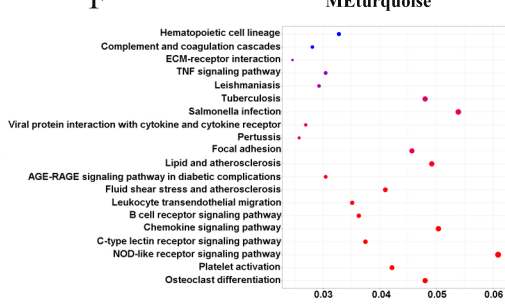

G

MEgrey

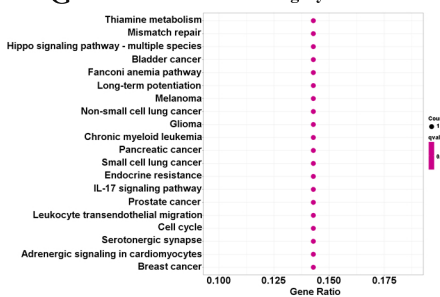

Supplement: Supplementary file 4 — Figure S4: KEGG analysis of DEGs in each co‐expression module. (a) Blue module; (b) red module; (c) green module; (d) yellow module; (e) brown module; (f) turquoise module; (g) gray module. [file FSN3-14-e71483-s004.pdf]

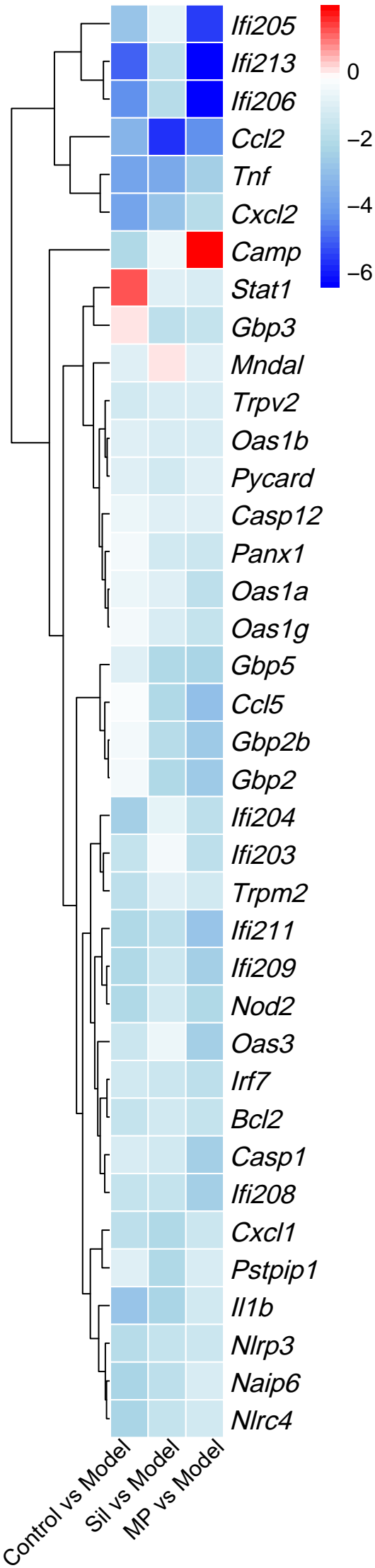

Supplement: Supplementary file 5 — Figure S5: Expression of genes related to the NOD‐like receptor signaling pathway. [file FSN3-14-e71483-s007.pdf]
